# Draft Genome Sequence of *Erwinia oleae*, a Bacterium Associated with Olive Knots Caused by *Pseudomonas savastanoi* pv. savastanoi

**DOI:** 10.1128/genomeA.01308-14

**Published:** 2014-12-11

**Authors:** Chiaraluce Moretti, Chiara Cortese, Daniel Passos da Silva, Vittorio Venturi, Giuseppe Firrao, Roberto Buonaurio

**Affiliations:** a Dipartimento di Scienze Agrarie, Alimentari e Ambientali, Università degli Studi di Perugia, Perugia, Italy; b International Centre for Genetic Engineering and Biotechnology, Trieste, Italy; c Dipartimento di Scienze Agrarie e Ambientali, Università degli Studi di Udine, Udine, Italy

## Abstract

*Erwinia oleae* is a nonpathogenic bacterial species isolated from olive knots caused by *Pseudomonas savastanoi* pv. savastanoi. Since the presence of *E. oleae* in the knots increases disease severity, interspecies interactions with the pathogen are hypothesized. Here, we report the first draft genome sequence of the *E. oleae* type strain.

## GENOME ANNOUNCEMENT

Knots caused by *Pseudomonas savastanoi* pv. savastanoi in olive trees provide an interesting niche for studying bacterial multispecies interactions. In fact, many bacterial species live inside the olive knots, which mainly belong to the genera *Pantoea*, *Pectobacterium*, *Erwinia*, and *Curtobacterium*, as revealed by metagenomic analysis (1). Among them, two *Erwinia* species, *Erwinia toletana* (2), whose genome has been recently sequenced (3), and *Erwinia oleae* (4) are well characterized and are responsible for the increase in olive knot disease severity when co-inoculated with the pathogen in the olive plants (5, 6). To better understand the molecular basis of interaction between *P. savastanoi* pv. savastanoi and *E. oleae* during knot development, we sequenced the genome of the type strain DAPP-PG 531 of *E. oleae*, isolated in Italy from an olive knot (4).

The genomic DNA for sequencing was prepared using the Nextera DNA sample preparation kit (Illumina), according to the manufacturer’s instructions. Sequencing was performed on an IlluminaMiSeq platform using indexed paired-end 250-nucleotide v2 chemistry. The sequencing produced an output of 778,712 reads representing approximately 40-fold coverage of the genome. Assembly, made by Edena assembler (7), yielded 185 contigs with a maximum length of 202 kbp and *N*_50_ of 50 kbp, assuming a genome size of 4.74 Mb. The G+C content is 54.7%, which is within the range reported for members of the genus *Erwinia* including *E. toletana*, the phylogenetically most closely related species of the genus also isolated from olive knots (2, 4).

Annotation of the *E. oleae* draft genome sequence conducted on the RAST (8) server predicted a total of 4,619 candidate protein coding-genes with 1,266 (27.4%) annotated as hypothetical proteins. The assembly predicted to contain 73 tRNA and 32 rRNA sequences. Comparative genome analysis was performed with the *E. toletana* DAPP-PG 735 genome (GenBank accession no. AOCZ01000000) using MUMmer (9). The results showed that 18% of the *E. oleae* genome aligned with *E. toletana* with an average of 85% of identity.

Analysis of the *E. oleae* genome revealed the presence of two canonical quorum-sensing systems. This report presents the first draft genome sequence of an *E. oleae* strain.

### Nucleotide sequence accession numbers.

This whole-genome shotgun project has been deposited at DDBJ/EMBL/GenBank under the accession no. JNVB00000000. The version described in this paper is version JNVB00000000.1.
